# Associations between non-traditional lipid measures and risk for type 2 diabetes mellitus in a Chinese community population: a cross-sectional study

**DOI:** 10.1186/s12944-016-0239-y

**Published:** 2016-04-05

**Authors:** Qiaofeng Song, Xiaoxue Liu, Anxin Wang, Youxin Wang, Yong Zhou, Wenhua Zhou, Xizhu Wang

**Affiliations:** Department of Cardiology, Tangshan People’s Hospital, North China University of Science and Technology, No.65 Shengli Road, Lunan District, Tangshan, 063000 China; Department of Epidemiology and Health Statistics, School of Public Health, Capital Medical University, Beijing, China; Department of Neurology, Beijing Tiantan Hospital, Capital Medical University, Beijing, China; Beijing Municipal Key Laboratory of Clinical Epidemiology, School of Public Health, Capital Medical University, Beijing, 100050 China

**Keywords:** Lipids, Non-HDL cholesterol, type 2 diabetes

## Abstract

**Background:**

This study investigated associations between type 2 diabetes mellitus and non-traditional lipid measures (total cholesterol (TC)/high-density lipoprotein cholesterol (HDL-C), triglycerides (TG)/HDL-C, and non-HDL-C).

**Methods:**

We conducted a community-based, cross-sectional study of 9 078 participants aged 18 years or older (4 768 men and 4 310 women) who lived in the Jidong community, Tangshan, China. The adjusted odds ratios for type 2 diabetes were calculated for every standard deviation change in TC, log-transformed TG, HDL-C, LDL-C, non-HDL-C, TC/HDL-C, and log-transformed TG/HDL-C using multivariate logistic regression analysis. Receiver operating characteristic (ROC) curve analysis was used to define the points of maximum sum of sensitivity and specificity for each lipid measure as a predictor for type 2 diabetes.

**Results:**

Prevalence of type 2 diabetes was 6.29 %. Higher TC, TG, LDL-C, non-HDL-C, TC/HDL-C, and TG/HDL-C, and lower HDL-C levels were individually associated with type 2 diabetes in multivariate analyses (all *P <* 0*.*05). TC/HDL-C was superior at discriminating between participants with and without type 2 diabetes compared with LDL-C (comparing ROC: *P* < 0.001), HDL-C (*P* < 0.001), TG (*P* = 0.012), TC (*P* < 0.001), non-HDL-C (*P* = 0.001), and TG/HDL-C (*P* = 0.03). The cutoff point for TC/HDL-C was 1.30 mmol/L in this population from the Jidong community. Sensitivity and specificity values for TC/HDL-C were 0.77 and 0.53, respectively.

**Conclusions:**

TC/HDL-C is associated with type 2 diabetes and is superior to LDL-C and HDL-C as a risk marker in this population.

## Background

The prevalence of type 2 diabetes mellitus is increasing globally. In the United States, approximately 18.8 million people were diagnosed with diabetes mellitus in 2010 [1]. The China National Diabetes and Metabolic Disorders Study reported that there were 92.4 million adults with diabetes from June 2007 to May 2008 in China [2]. However, there was also about 148.2 million adults with prediabetes, defined as impaired fasting glucose and/or impaired glucose tolerance. Identification of risk factors for the development of type 2 diabetes is urgently needed to better understand and prevent this public health epidemic in China.

Previous studies have confirmed that type 2 diabetes can be prevented by identifying and intervening in the development of risk factors [3–5]. Studies have shown that dyslipidemia is one of the known risk factors for type 2 diabetes [6, 7]. Traditional lipid measures, including total cholesterol (TC), low-density lipoprotein cholesterol (LDL-C), and triglycerides (TG), are well documented in their association with the incidence of type 2 diabetes [6, 8]. Additionally, evidence indicates that non-high-density lipoprotein cholesterol (non-HDL-C) is a superior marker for the development of vascular disease compared with LDL-C, the traditional lipid measure [9–12]. Other studies have reported that lipid ratios, such as TC/HDL-C, have shown a predictive value for cardiovascular disease [13, 14].

Ley et al. reported that non-HDL-C was associated with the incidence of type 2 diabetes and was superior to LDL-C as a risk predictor in 606 diabetes-free participants [15]. Another prospective study reported that lipid ratios (TG/HDL-C and TC/HDL-C) were associated with the incidence of type 2 diabetes among Iranians [16]. There is a lack of data on associations between non-traditional lipid measures (TC/HDL-C, TG/HDL-C, and non-HDL-C) and type 2 diabetes in China. The current study investigated the association between non-traditional lipid measures with type 2 diabetes and compared their predictive significance to those of traditional lipid variables, including TC, LDL-C, and TG, in a population from the Jidong community, China. We hypothesized that non-traditional lipid measures (TC/HDL-C, TG/HDL-C, and non-HDL-C) are associated with type 2 diabetes and are superior to LDL-C and HDL-C as risk markers in this population.

## Methods

### Study design and population

From July 2013 to August 2014, 9 078 residents of the Jidong community aged 18 years and older were invited to participate in the study. The community is geographically located in Tangshan, which is a modern industrial city located in the central section of the circulating Bohai Sea Gulf region of China and mainly comprises employees of Jidong Oilfield Inc. After excluding participants with incomplete information on type 2 diabetes and lipid measurement data, 8 829 participants were included in the study. At baseline, physical examinations and surveys were conducted by trained medical professionals from medical centers of Jidong Oil Field Inc.

The study was approved by the Ethics Committee of Jidong Oilfield Inc. Medical Centers, and signed informed consent was obtained from all participants.

### Assessment of potential covariates

A questionnaire was used to obtain data on age, sex, marital status, personal monthly income, smoking, drinking, physical activity, education level, and history of diseases (hypertension, type 2 diabetes, stroke, cancer, myocardial infarction). Questionnaires were administered in person by research doctors.

Anthropometric measurements, including height, weight, body mass index (BMI), and blood pressure, were measured by three trained doctors and nurses. Height was measured to the nearest 0.1 cm using a tape measure. Weight was measured to the nearest 0.1 kg using calibrated platform scales. BMI was calculated as body weight (kg) divided by the square of height (m^2^). Blood pressure was measured using a mercury sphygmomanometer. Two readings for systolic blood pressure and diastolic blood pressure were taken at 5-min intervals with participants resting in a chair during between measurements. The average of the two readings was used for analyses. If the two measurements differed by more than 5 mmHg, an additional reading was taken, and the average of the three readings was used [17]. Hypertension was defined based on a personal history of hypertension, systolic blood pressure ≥140 mmHg, diastolic blood pressure ≥90 mmHg, or currently taking antihypertensive medication prescribed by a physician [18].

### Biochemical index

Blood samples were drawn by trained phlebotomists from participants after an overnight fast for 8–12 h. Venous blood samples in tubes containing ethylenediaminetetraacetic acid trisodium salt hydrate were immediately placed on ice after antecubital venipuncture. Fasting blood glucose (FBG) was measured using the hexokinase/glucose-6-phosphate dehydrogenase method [19]. TC was measured using the endpoint test method [20]. HDL-C and LDL-C levels were measured using the direct test method [21], and TG was measured using the GPO method [22] (inter-assay coefficient of variation: <10 %; Mind Bioengineering Co. Ltd, Shanghai, China). All biochemical variables were measured using an auto analyzer (Olympus AU400; Olympus, Tokyo, Japan) at the central laboratory at Jidong Oilfield Hospital. Non-HDL-C levels were determined by subtracting serum HDL-C levels from TC [11, 15, 16].

### Diagnostic criteria for type 2 diabetes

Type 2 diabetes was diagnosed if the participant was undergoing treatment with insulin or oral hypoglycemic agents, if FBG levels were ≥7.0 mmol/L, or if the participant had a personal history of type 2 diabetes [2].

### Statistical analysis

Statistical analyses were performed using SAS software, version 9.4 (SAS Institute, Cary, NC, USA). Data are presented as mean ± standard deviation (SD) for continuous variables and as frequencies and percentages for categorical variables. Because TG and TG/HDL-C had a skewed distribution, they are presented as medians (interquartile ranges). The Student’s *t*-test was used for non-paired samples for comparisons of normally distributed parameters, and the Wilcoxon test was used for comparisons of non-parametric variables. The chi-squared test was used for comparisons of categorical variables.

Multiple logistic regression analysis was conducted to evaluate associations between lipid measures with type 2 diabetes. The adjusted odds ratio (OR) per 1 SD increase in the corresponding lipid variable and 95 % confidence intervals (CI) were calculated. The area under the receiver operating characteristic (ROC) curve (95 % CI) was used to compare the ability of different lipid variables in discriminating participants with or without type 2 diabetes.

To determinate appropriate cutoff values of each lipid measure for predicting type 2 diabetes, ROC curve analysis was also used to estimate the variable’s sensitivity and specificity. The cutoff point of each lipid variable was assessed by the minimum value of √ (1-sensitivity)^2^ + (1-specificity)^2^, which represent the maximum sum of sensitivity and specificity [23].

All statistical tests were two-sided. A *P-*value <0.05 was considered statistically significant.

## Results

Of the 8 829 participants (4 625 men and 4 204 women; mean age of 42 years), 555 (6.29 %) were diagnosed with type 2 diabetes. Compared with those without type 2 diabetes, patients with type 2 diabetes had higher TC/HDL-C, TG/HDL-C, non-HDL-C, TC, TG, LDL-C, BMI, and FBG, and were more likely to have hypertension at baseline (all *P <* 0*.*001) (Table 1).

**Table 1:** Baseline characteristics of study participants with and without type 2 diabetes

Characteristics	Diabetes	Without diabetes	*P-*value
No.	555	8274	
Age (years)	54.40 ± 10.98	41.12 ± 12.73	<0.001
Gender, male, *n* (%)	354(63.78)	4271(51.62)	<0.001
Married status (yes)	547(98.56)	7605(91.91)	<0.001
Income, ¥/month			<0.001
≤¥3000	293(54.06)	3020(37.09)	
¥3001–5000	219(40.41)	4495(55.21)	
≥¥5001	30(5.54)	627(7.70)	
Education level			<0.001
Illiteracy/primary	50(9.01)	267(3.23)	
Middle school	335(60.36)	2790(33.72)	
College/University	170(30.63)	5217(63.05)	
Drinking (yes)	196(35.31)	2743(33.15)	<0.001
Smoking (yes)	177(31.95)	2121(25.64)	<0.001
Physical activity (active)	351(63.24)	4216(50.95)	<0.001
BMI	26.74 ± 3.54	24.35 ± 3.67	<0.001
Blood pressure			
Systolic blood pressure (mmHg)	142.02 ± 21.19	125.26 ± 17.98	<0.001
Diastolic blood pressure (mmHg)	87.61 ± 13.53	80.44 ± 12.85	<0.001
Hypertension, yes	379(68.29)	2318(28.02)	<0.001
Lipid profile			
LDL-C (mmol/L)	2.75 ± 0.65	2.45 ± 0.60	<0.001
HDL-C (mmol/L)	1.10 ± 0.25	1.20 ± 0.27	<0.001
TG (mmol/L)^a^	1.71(1.16–2.63)	1.21(0.84–1.84)	<0.001
TC (mmol/L)	4.81 ± 0.96	4.42 ± 0.87	<0.001
Non-HDL-C (mmol/L)	3.70 ± 0.90	3.22 ± 0.85	<0.001
TG/HDL-C^a^	1.58(1.00–2.66)	1.04(0.65–1.72)	<0.001
TC/HDL-C	4.50 ± 1.13	3.83 ± 1.02	<0.001
Fasting glucose (mmol/L)	8.24 ± 2.48	5.04 ± 0.50	<0.001

*BMI* body mass index, *HDL-C* high-density lipoprotein cholesterol, *LDL-C* low-density lipoprotein cholesterol, *Non-HDL-C* non-high-density lipoprotein cholesterol, *TC* total cholesterol, *TG* triglycerides

^a^Medians (25th–75th percentile)

OR of 1 SD change in each lipid marker or index are listed in Table 2. In the fully adjusted model, there were positive correlations between TC, TG, non-HDL-C, TG/HDL-C, and TC/HDL-C with type 2 diabetes, and 1 SD increase in each of these lipids increased risk for type 2 diabetes by 13 %, 38 %, 23 %, 45 %, and 42 %, respectively (Table 2). The positive correlations between each lipid marker with type 2 diabetes persisted when participants with hypertension were further excluded in the sensitivity analyses (Table 2).

**Table 2:** Logistic regression analysis of associations between lipids and type 2 diabetes

		Odds ratio (OR) (95 % CI)			Sensitivity analyses^d^
	SD	Model 1^a^	Model 2^b^	Model 3^c^	Odds ratio (95 % CI)
LDL-C (mmol/L)	0.6086	1.57(1.45–1.70)	1.21(1.10–1.33)	1.14(1.04–1.25)	1.21(1.03–1.43)
HDL-C (mmol/L)	0.2696	0.68(0.61–0.75)	0.68(0.61–0.76)	0.74(0.66–0.83)	0.66(0.54–0.81)
TG (mmol/L)^e^	0.5976	1.68(1.56–1.82)	1.49(1.36–1.63)	1.38(1.25–1.53)	1.55(1.31–1.84)
TC (mmol/L)	0.8792	1.50(1.39–1.63)	1.18(1.08–1.29)	1.13(1.03–1.24)	1.18(1.00–1.38)
Non-HDL-C (mmol/L)	0.8595	1.66(1.54–1.80)	1.31(1.20–1.44)	1.23(1.12–1.35)	1.32(1.12–1.55)
TG/HDL-C^f^	0.7256	1.71(1.58–1.85)	1.56(1.42–1.72)	1.45(1.31–1.60)	1.64(1.38–1.96)
TC/HDL-C	1.0356	1.70(1.57–1.83)	1.52(1.39–1.66)	1.42(1.30–1.57)	1.59(1.37–1.85)

Odds ratio (OR) (95 % CI) per 1 SD change. *HDL-C* high-density lipoprotein cholesterol, *LDL-C* low-density lipoprotein cholesterol, *Non-HDL-C* non-high-density lipoprotein cholesterol, *SD* standard deviation, *TC* total cholesterol, *TG* triglycerides

^a^Unadjusted

^b^Adjusted for age, sex, marital status, personal monthly income level, education level, smoking, drinking, physical activity, and hypertension

^c^Additionally adjusted for BMI

^d^Adjusted for model 3 and further excluded individuals with hypertension

^e^Log-transformed TG used

^f^Log-transformed TG/HDL-C used

Table 3 shows the ROC curve and cutoff points for lipid measures for the prediction of type 2 diabetes with their corresponding specificity and sensitivity. Cutoff points for LDL-C, HDL-C, TG, non-HDL-C, TG/HDL-C, and TC/HDL-C to predict type 2 diabetes were 1.20, 1.18, 1.23, 1.19, 1.24, and 1.30 mmol/L, respectively.

**Table 3:** ROC curve for predicting diabetes and cutoff points for maximum sum of sensitivity and specificity

	ROC (95 % CI)	Cut point	Sensitivity (%)	Specificity (%)	ROC (95 % CI)^a^
LDL-C (mmol/L)	0.632 (0.608–0.655)	1.20	0.60	0.61	0.648 (0.608–0.687)
HDL-C (mmol/L)	0.603 (0.579–0.627)	1.18	0.65	0.53	0.632 (0.589–0.674)
TG (mmol/L)	0.659 (0.637–0.683)	1.23	0.58	0.65	0.669 (0.629–0.708)
TC (mmol/L)	0.621 (0.597–0.646)	1.19	0.53	0.66	0.626 (0.585–0.668)
Non-HDL-C (mmol/L)	0.656 (0.634–0.679)	1.23	0.61	0.62	0.673 (0.635–0.712)
TG/HDL-C	0.666 (0.643–0.688)	1.24	0.87	0.37	0.682 (0.644–0.720)
TC/HDL-C	0.684 (0.663–0.705)	1.30	0.77	0.53	0.717 (0.680–0.754)

*HDL-C* high-density lipoprotein cholesterol, *LDL-C* low-density lipoprotein cholesterol, *Non-HDL-C* non-high-density lipoprotein cholesterol, *TC* total cholesterol, *TG*, triglycerides. Cut points are presented as mmol/L

TC/HDL-C was a better predictor than LDL-C (comparing ROC: *P* < 0.001), HDL-C (*P* < 0.001), TG (*P* = 0.012), TC (*P* < 0.001), non-HDL-C (*P* = 0.001), and TG/HDL-C (*P* = 0.03)

^a^Further excluded individuals with hypertension

Comparing ROC curves, TC/HDL-C was a more superior discriminant of participants with and without type 2 diabetes than LDL-C (comparing ROC: *P* < 0.001), HDL-C (*P* < 0.001), TG (*P* = 0.012), TC (*P* < 0.001), non-HDL-C (*P* = 0.001), and TG/HDL-C (*P* = 0.03).

The significant positive association between each lipid marker with type 2 diabetes and the trend seen by comparing ROC curves for each lipid marker persisted when participants with hypertension were further excluded in the sensitivity analyses (Table 3).

## Discussion

In this study, TC/HDL-C was found to be associated with type 2 diabetes, and was a superior marker or index for discriminating individuals with and without type 2 diabetes compared with LDL-C, HDL-C, or non-HDL-C in this population from the Jidong community, China.

Although the association between lipid ratios and incident vascular disease is well documented [10, 13, 14], data on associations between TC/HDL-C and TG/HDL-C with type 2 diabetes are limited. Our study suggests that higher TC/HDL-C or TG/HDL-C is associated with an increased risk for type 2 diabetes. TC/HDL-C is an indirect estimate of LDL-C particle number, and the LDL-C particle number is closely related to atherosclerosis and cardiometabolic risk compared with LDL-C [9]. Similar to the findings of the current study, a previous prospective study reported that adjusted TC/HDL-C is associated with the incidence of type 2 diabetes among Iranian men (OR: 1.44; 95 % CI: 1.22–1.69) and women (OR: 1.37; 95 % CI: 1.21–1.55) [16], and adjusted TG/HDL-C is associated with the incidence of type 2 diabetes among Iranian men (OR: 1.44; 95 % CI: 1.25–1.65) and women (OR: 1.78; 95 % CI: 1.53–2.06) [16]. Ley et al. also reported that TC/HDL-C is associated with the incidence of type 2 diabetes in non-Aboriginal populations [15]. Kimm et al. reported that lipid ratios, including TC/HDL-C and TG/HDL-C, are associated with insulin resistance and might be used as integrated lipid values [24].

In the current study, areas under ROC curves for each lipid measure (LDL-C, HDL-C, TG, TC, non-HDL-C, TG/HDL-C, and TC/HDL-C) were 0.632, 0.603, 0.659, 0.621, 0.656, 0.666, and 0.684, respectively, suggesting that TC/HDL-C might be a superior predictor of type 2 diabetes compared with LDL-C. As mentioned, Hadaegh et al. [16] also reported lipid ratios were superior in their association with type 2 diabetes in Iranian men and women. However, in another prospective study carried out by Ley et al. [15], the researchers reported that non-HDL-C was associated with the incidence of type 2 diabetes and was superior to LDL-C as a risk predictor in that population. The differences may arise from differences in ethnicities of the studied populations. Participants in the current study are mainly Han-ethnic Chinese (97.2 %), probably having different diverse socioeconomic factors and lifestyles compared with other ethnic populations.

In the data analysis in the current study, ROC analysis was used to calculate the cutoff points of lipid variables based equally on weighted sensitivity and specificity, hence these cutoff points have low sensitivity for predicting diabetes in clinical practice. Additionally, these points may not be the optimal cutoffs in clinical settings because sensitivity vs. specificity should be weighed against many other factors, such as the seriousness of the illness, how invasive or feasible it might be, the test used for first evaluation, and finally how often the test should be performed [25].

Several limitations should be considered when interpreting the results of the current study. First, type 2 diabetes was diagnosed based on a single FBG level without an oral glucose tolerance test [15] for further diagnosis. It is therefore possible that patients with type 2 diabetes might have been missed. This would result in the impact between increased lipid ratios and type 2 diabetes being underestimated. However, the same method for diagnosing type 2 diabetes with a single FBG measurement was used in previous epidemiological studies (the Framingham Heart Study [26] and the Kailuan study [19]). Second, inclusion of individuals who had a higher proportion of hypertension at baseline might influence the results of the association. However, with sensitivity analyses, these people were further excluded, and the significant positive associations between type 2 diabetes with each lipid marker and the trend seen when comparing the C-statistics for each lipid marker were consistent. Third, all participants are from the Jidong community, and have a relatively higher income status and have attained higher education compared with the general Chinese population. Therefore, generalizations about the entire Chinese population cannot be drawn from the results. Finally, this study was a cross-sectional study, which limits the ability to conclude a cause-effect association between lipid ratios and type 2 diabetes.

## Conclusions

The results of the current study suggest that TC/HDL-C strongly correlates with type 2 diabetes, and together with established independent lipid measures such as HDL-C and LDL-C in this population. The predicative significance of TC/HDL-C for type 2 diabetes is better than LDL-C or HDL-C in this Chinese population. The results support using TC/HDL-C information, which is becoming more readily available in clinical settings, for communicating the increased risk for cardiometabolic disease progression at an early stage, before the onset of diabetes. It is suggested that further studies are required to establish the utility of TC/HDL-C in more populations before it can be incorporated into clinical decision making for the prevention of type 2 diabetes.

### Ethics approval and consent to participate

The study was approved by the Ethics Committee of Jidong Oilfield Inc. Medical Centers, and signed informed consent was obtained from all participants.

## Abbreviations

BMI: body mass index
CI: confidence interval
FBG: fasting blood glucose
HDL-C: high-density lipoprotein cholesterol
LDL-C: low-density lipoprotein cholesterol
OR: odds ratio
ROC: receiver operating characteristic
SD: standard deviation
TC: total cholesterol
TG: triglycerides
